# Effects of Limited Hydrolysis and High-Pressure Homogenization on Functional Properties of Oyster Protein Isolates

**DOI:** 10.3390/molecules23040729

**Published:** 2018-03-22

**Authors:** Cuiping Yu, Yue Cha, Fan Wu, Xianbing Xu, Ming Du

**Affiliations:** School of Food Science and Technology, National Engineering Research Center of Seafood, Dalian Polytechnic University, Dalian 116034, China; yucuiping1987@sina.com (C.Y.); m18840853913@163.com (Y.C.); 15536665462@163.com (F.W.); xianbingxu@dlpu.edu.cn (X.X.)

**Keywords:** oyster protein isolates, acidic treatment, pepsin, high-pressure homogenization, functional properties, structure

## Abstract

In this study, the effects of limited hydrolysis and/or high-pressure homogenization (HPH) treatment in acid conditions on the functional properties of oyster protein isolates (OPI) were studied. Protein solubility, surface hydrophobicity, particle size distribution, zeta potential, foaming, and emulsifying properties were evaluated. The results showed that acid treatment led to the dissociation and unfolding of OPI. Subsequent treatment such as limited proteolysis, HPH, and their combination remarkably improved the functional properties of OPI. Acid treatment produced flexible aggregates, as well as reduced particle size and solubility. On the contrary, limited hydrolysis increased the solubility of OPI. Furthermore, HPH enhanced the effectiveness of the above treatments. The emulsifying and foaming properties of acid- or hydrolysis-treated OPI significantly improved. In conclusion, a combination of acid treatment, limited proteolysis, and HPH improved the functional properties of OPI. The improvements in the functional properties of OPI could potentiate the use of oyster protein and its hydrolysates in the food industry.

## 1. Introduction 

In recent years, much attention has been paid to marine ingredients (proteins, polysaccharides, and pigments) from seaweed, microalgae, and marine animals in the food and animal feed industries; in particular, microalgal proteins have been reported to play important roles [1,2,3,4,5]. Shellfish are abundant in physiologically active components which play crucial roles in functional foods [6]. Shellfish contain a high essential amino acid/non-essential amino acid ratio [7]. Oyster is a common and popular shellfish because of its high nutritional quality and unique flavors. Oyster protein isolates (OPI) show good potential as a nutritional protein source; however, OPI is not yet widely used. Generally, the functional properties of native proteins are not good enough. Therefore, different technologies have been developed to improve important functional properties of proteins, such as solubility, emulsifying properties, foaming properties, and gel properties. In our previous study, it was found that high-pressure homogenization improved the solubility, emulsifying properties, and foaming properties of the mussel protein isolates [8].

In recent years, various food processing technologies have been widely applied to modify the functional properties of food protein, including heat treatments, enzymatic hydrolysis, pulsed electric fields, ultrasonic treatments, high pressure, and high-pressure homogenization (HPH). High pressure was reported to disrupt the quaternary and tertiary structure of globular proteins with relatively little influence on their secondary structure [9,10]; as a result, properties of proteins changed. Generally, the efficiency of a single modification is relatively low. Many studies have found that combined treatments can improve functional properties of food protein. For example, it has been reported that the emulsifying and foaming capacity of soybean protein significantly increased after combined treatments of limited hydrolysis with pepsin and HPH [11]. The combined treatments of limited hydrolysis with alcalase and HPH significantly improved the solubility and emulsification capability of glycinin [12]. Ultrasound pretreatment was found to markedly increase the emulsifying capability of papain-hydrolyzed soy protein isolate [13]. High pressure treatments enhance the hydrolysis degree of soybean whey proteins with trypsin, chymotrypsin, and pepsin, respectively [14].

The aim of this work was to explore the effects of limited proteolysis with pepsin and/or HPH in acid conditions on the structure and functional properties of OPI, and then find an eco-friendly processing method to expand its application.

## 2. Results and Discussion

### 2.1. Degree of Hydrolysis (DH) of OPI Hydrolysis with Different Ratios of Enzyme to Substrate (E/S)

**Figure 1:**
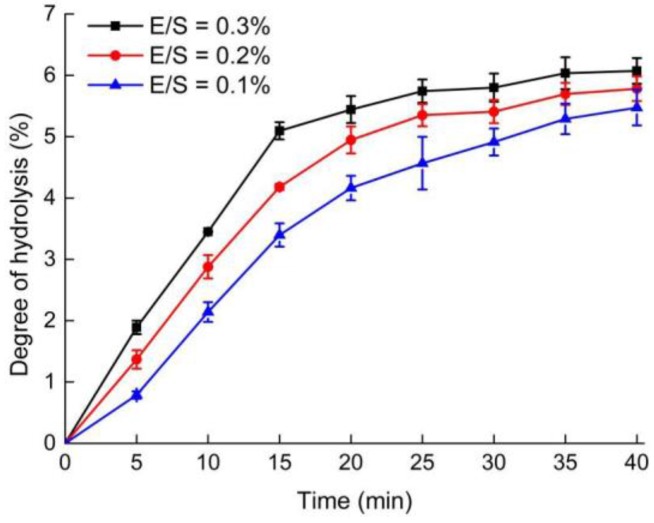
Degree of hydrolysis (DH) of oyster protein isolate (OPI) hydrolysis with different ratios of enzyme to substrate (E/S) (0.1%, 0.2%, 0.3%, *w*/*w*). The protein concentration of OPI was 10% (*w*/*v*).

The enzymatic modification of food proteins by controlled proteolysis can enhance their functional properties. A previous study found that the limited proteolysis products of the average DH of 1% to 10% have better functional properties than native proteins [15]. Thus, it is important to choose an optimal ratio of E/S to perform limited proteolysis. The hydrolysis of OPI with different ratios of E/S (0.1%, 0.2%, 0.3%, *w*/*w*) was carried out by the 2,4,6-trinitrobenzene sulfonic acid (TNBS) method (Figure 1). When E/S was 0.3%, hydrolysis progressed rapidly within the first 15 min and the DH of OPI increased to 5.1% ± 0.1%. Thereafter, the rate subsequently decreased. When E/S was 0.1% and 0.2%, the reaction rates were lower compared with that at an E/S ratio of 0.3%. This finding suggested that a larger E/S ratio resulted in shorter reaction time to reach a certain DH. Based on the above results, we concluded that an E/S ratio of 0.3%, with proteolysis for 15 min, was the optimal condition for the following experiments. These results were consistent with previous studies of soy protein isolate [16].

### 2.2. Composition and SDS-PAGE Analysis of Protein Isolates and Hydrolysates

**Figure 2:**
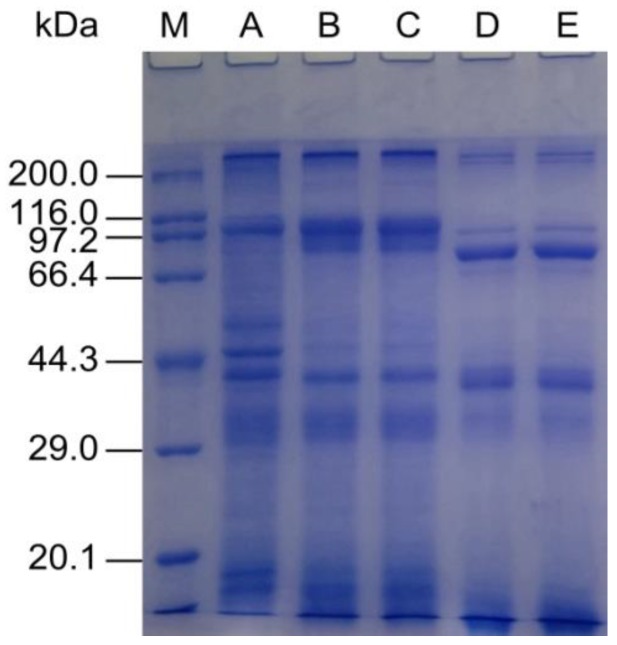
SDS-PAGE profiles of OPI and the modified samples. M: molecular weight marker; A: OPI; B: Acid treatment (AT); C: Acid treatment combined with high-pressure homogenization treatment (AT-HPH); D: Acid treatment combined with limited proteolysis treatment (AT-LP); E: Acid, limited proteolysis, and high-pressure homogenization treatment (AT-LP-HPH).

Contents of moisture, proteins, fat, carbohydrates, and ash in OPI were 3.0% ± 0.1%, 81.0% ± 2.5%, 2.0% ± 0.3%, 10.0% ± 1.7%, and 4.0% ± 0.5%, respectively. SDS-PAGE was performed to evaluate the effects of various treatments on protein samples (Figure 2). As for control groups (lane A), SDS-PAGE analysis showed that OPI contained several bands, and the approximate molecular weights were 246.0, 107.0, 52.0, 45.0, 41.0, 33.0, 19.0, and 17.0 kDa. Compared with lane A, a band of approximately 107.0 kDa became weaker and another band of approximately 45.0 kDa disappeared in the rest of the lanes. These indicated that acid treatment induced protein unfolding and degradation [17]. Compared with OPI and the acid treatment group (AT), the acid treatment combined with limited proteolysis treatment (AT-LP) groups contained more polypeptides of low molecular weight, and intensities of the three main bands were much weaker (lane D). This suggested that some subunits of OPI degraded into smaller fragments after proteolysis. Furthermore, lane C and lane E showed no difference from lane B and lane D, respectively, which suggested that HPH treatment did not result in any changes in the protein bands. This phenomenon was similar to our previous study of high-pressure homogenized mussel protein isolate [8].

### 2.3. Protein Solubility 

Solubility is an essential functional property of protein. High solubility is pivotal for many protein-based formulations, since solubility affects other properties, especially foaming and emulsification [18]. As shown in Table 1, solubility was relatively low near the isoelectric point (pH 4–6). When pH was adjusted to 3 or more than 7, solubility was relatively high. Samples of the AT-LP and acid, limited proteolysis, and high-pressure homogenization treatment group (AT-LP-HPH) showed higher solubility between pH 4 and 6, which might be because the limited proteolysis treatment produced peptides of low molecular weight, and exposed ionizable amino and carboxyl groups increased correspondingly [15,19,20]. At a pH around the isoelectric point, an increase of solubility was found, implying the additional effect of limited proteolysis. In addition, AT had the lowest solubility at all pH values. This phenomenon can be explained by the fact that oyster proteins unfolded and hydrophobic residues were exposed after acid treatment [20]. From pH 6 to 11, the solubility of all of the samples showed a progressive increase and the highest solubility was found in AT-LP-HPH samples at pH 10, indicating that combined modification could improve the solubility. Furthermore, samples treated by HPH, including the acid treatment combined with high-pressure homogenization group (AT-HPH) and the AT-LP-HPH group, showed higher solubility than the unhomogenized samples (AT and AT-LP), which demonstrated that HPH increased the solubility of protein at 60 MPa. A previous study also found that HPH improved the water solubility of myofibrillar protein [21].

At pH 7.0, the solubility of OPI, AT, AT-HPH, AT-LP, and AT-LP-HPH was 27.1% ± 0.7%, 19.9% ± 0.8%, 20.6% ± 1.1%, 27.4% ± 0.9%, and 31.7% ± 1.4%, respectively. The improved solubility of the limited hydrolyzed samples may be due to the destruction of the compact structure and insoluble aggregates. As a result, more charged and polar groups became exposed, and soluble aggregates formed [15]. The decreased solubility of acid-treated samples may be because OPI was easily denatured under low pH conditions [22]. This indicated that HPH combined with limited hydrolysis improved the solubility of OPI.

### 2.4. Particle Size Distribution and Zeta Potential 

**Figure 3:**
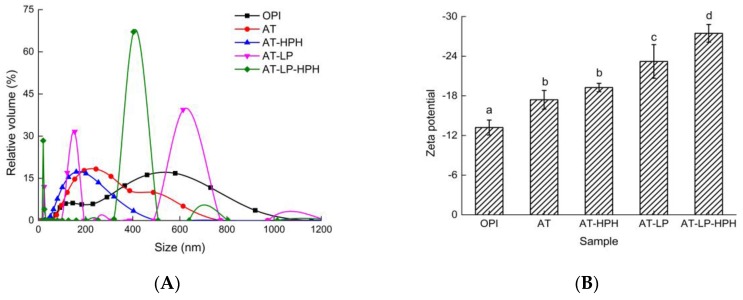
Particle size distribution (**A**) and zeta potential (**B**) of OPI and the modified samples. Different letters represent significant differences at *p* < 0.05.

Figure 3A shows the particle size distribution of protein samples at pH 7.0. The particle size of OPI was mainly distributed in the range of 100–1200 nm. After acid treatment, the particle size of AT was reduced to 70–800 nm. This indicated that proteins dissociated and produced small molecule groups under acid conditions. In addition, HPH combined with acid treatment resulted in a more uniform particle size distribution. After 15 min of limited hydrolysis, it is obvious that the particle size was reduced and the suspension system became more uniform. This may be a result of the hydrolysis of some proteins [23]. Moreover, HPH at 60 MPa showed the coordination efficiency with hydrolysis treatment. A previous study of egg white protein drew a similar conclusion that HPH is a facile mechanical method for reducing particle size [24].

Zeta potential is a dependable indicator of membrane surface charges, which reflects the stability of colloidal suspension [25]. Figure 3B shows the zeta potential of OPI and the modified samples at pH 7.0. The absolute zeta potential of the untreated OPI is low, indicating that the electrostatic repulsion among protein particles was weak. After acid treatment, it was observed that the absolute zeta potential significantly increased, indicating that more electrostatic repulsion between particles formed and the stability of protein solution increased. In addition, the high absolute zeta potential of AT-LP represented the stability of aqueous suspensions, which might be due to the dissociation of an amino acid group, resulting in changes of protein charge [26]. Furthermore, HPH-treated samples (AT-HPH and AT-LP-HPH) showed higher absolute zeta potential, which indicated that HPH treatment could promote the stability of OPI aqueous suspensions. This was consistent with a previous report which showed that the absolute zeta potential of myofibrillar proteins from chicken breast increased after HPH [27].

### 2.5. Surface Hydrophobicity

As shown in Table 2, acid treatment significantly increased *H*_0_ compared with that of OPI. This phenomenon can be explained by the fact that proteins unfolded after acid treatment, resulting in the exposure of hydrophobic groups. As for the AT-LP groups, *H*_0_ was lower compared with that of OPI. In general, protein hydrophobicity depends on experimental conditions, protein characteristics, and enzyme specificity [15]. It has been found that the limited hydrolysis of glycinin with alcalase increased the exposure of hydrophobic groups [12]. In contrast, a previous study also reported that the hydrophobicity of soy protein was reduced after hydrolysis with alkaline protease and esterase. This reduction might be attributed to greater protein flexibility and more exposed hydrophilic groups caused by hydrolysis [28]. Furthermore, HPH-treated samples such as AT-HPH and AT-LP-HPH showed higher *H*_0_ than that of AT and AT-LP, which indicated that more hydrophobic groups were exposed because of high pressure and shear forces after HPH treatment. This was similar to a previous report which showed that HPH treatment strengthened the formation of surface hydrophobicity of myofibrillar from chicken [12].

### 2.6. Foaming Properties 

**Figure 4:**
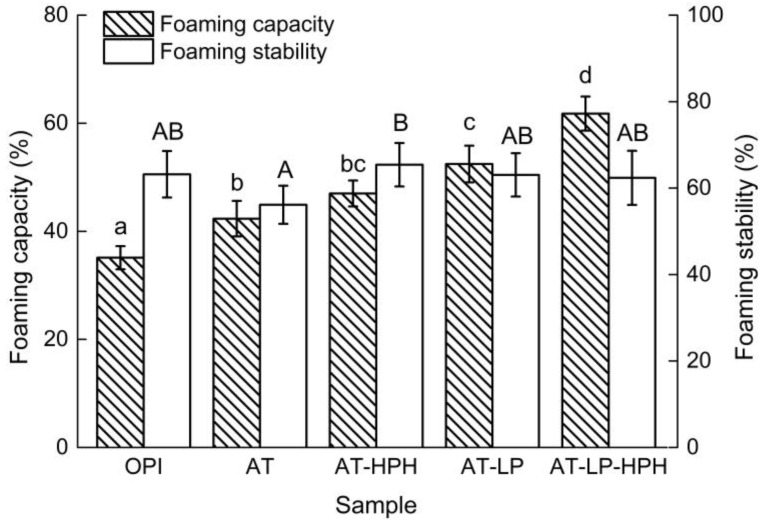
Foaming properties of OPI and the modified samples. Different lowercase and uppercase letters represent significant differences at *p* < 0.05.

Foaming properties depend on the degree of denaturation, type of protein, temperature, pH, and whipping methods [15]. A previous study reported that enzymatic hydrolysis may improve foaming capacity (FC) [29]. As shown in Figure 4, the FC of all of the modified samples significantly increased, indicating that treated OPI transferred to air-water interface more slowly and surface tension decreased [30]. The FC of AT increased from 35.1% ± 2.1% to 42.3% ± 3.3%, which might be due to high *H*_0_. After limited hydrolysis, the FC of AT-LP significantly increased to 52.4% ± 3.4%. This might be because the peptides produced by hydrolysis were easier to move to the liquid-air interface and change the conformational structure to form a protein film enclosing air [31]. As more flexible and low molecular weight aggregates were observed after combination treatment, the FC of AT-LP-HPH was the highest, up to 61.8% ± 3.2%. However, the FC of AT-HPH was close to that of AT, indicating that HPH in acid conditions had no effect on foaming capacity. As for foaming stability (FS), there was no significant change in the modified samples. The FS of AT was the lowest, which was probably because the adsorption film formed by covalent and non-covalent bonds could not prevent the re-aggregation of air bubbles. Compared with AT, the FS of AT-HPH was higher, which resulted from the exposure of hydrophobic groups after HPH.

### 2.7. Emulsifying Properties 

**Figure 5:**
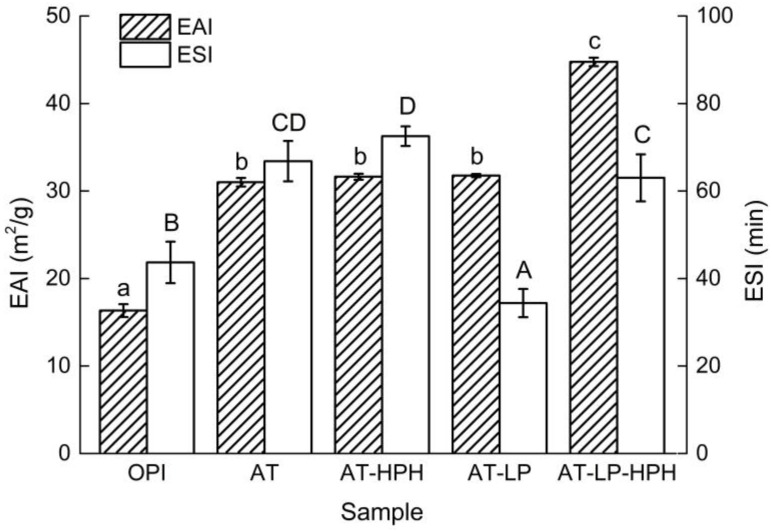
Emulsifying properties of OPI and the modified samples. Different lowercase and uppercase letters represent significant differences at *p* < 0.05.

Proteins are good emulsifiers due to their amphipathic nature [32]. Previous studies have found that appropriate enzyme and DH improved the emulsifying properties of proteins, and the optimum DH was relatively low (2–10%) [33,34]. Figure 5 shows the emulsifying properties of OPI and the modified samples. Compared with OPI, the emulsifying activity index (EAI) of AT increased significantly. This indicated that proteins were more likely to form an interfacial membrane after acid treatment, thereby promoting the dispersion of oil droplets more strongly than that observed for OPI. This finding was consistent with a previous study, which found that the foaming and emulsifying properties of soybean glycinin significantly improved after acid treatment [35]. In addition, the emulsifying stability index (ESI) of AT increased significantly compared with native OPI after acid treatment. This phenomenon can be attributed to the exposure of hydrophobic groups. The EAI and ESI of AT-HPH were close to that of AT, indicating that HPH treatment in acid conditions had no effect on EAI and ESI. Furthermore, the EAI of AT-LP was higher than that of OPI, but the ESI was lower than that of OPI. The increase of EAI corresponded to the increase of solubility and the decrease of particle size after limited hydrolysis. The degradation of protein molecules resulted in an increasing large number of peptide units at the oil/water interface, which is more conducive to emulsion formation. The decreased ESI of AT-LP might be because the resultant polypeptides after hydrolysis were too small to stabilize the oil/water interface. As for AT-LP-HPH, both the EAI and ESI increased significantly, which might be because the hydrophobic and electrostatic interactions of protein were disrupted after high pressure treatment. A previous study of sweet potato protein also showed that HPH treatment could improve the emulsifying properties [36].

## 3. Materials and Methods

### 3.1. Chemical Reagents

Fresh oysters (Magallana gigas) were purchased from Changxing market (Dalian, China). Pepsin (400 units/mg protein, Sigma, St. Louis, MO, USA) was from porcine gastric mucosa. The oil used for the emulsification properties measurement was corn oil (Xiwang Food Co., Ltd., Yantai, China). Bromophenol blue sodium salt (BPB), 2,4,6-trinitrobenzene sulfonic acid (TNBS), β-mercaptoethanol, acrylamide, and *N*,*N*′-Methylene-bis (acrylamide) were purchased from Sigma Chemical Co. (St. Louis, MO, USA). All other chemicals and reagents used in this study were of analytical grade.

### 3.2. Determination of Composition of OPI

Protein content was determined by the Kjeldhal method. Moisture content was determined by the oven-dry method [37]. Carbohydrates content was determined by the anthrone colorimetry method [38]. The solvent extraction method was used for fat measurement [39], and the dry ashing method was used for ash measurement [40].

### 3.3. Preparation of Protein Isolates and Hydrolysates

**Figure 6:**
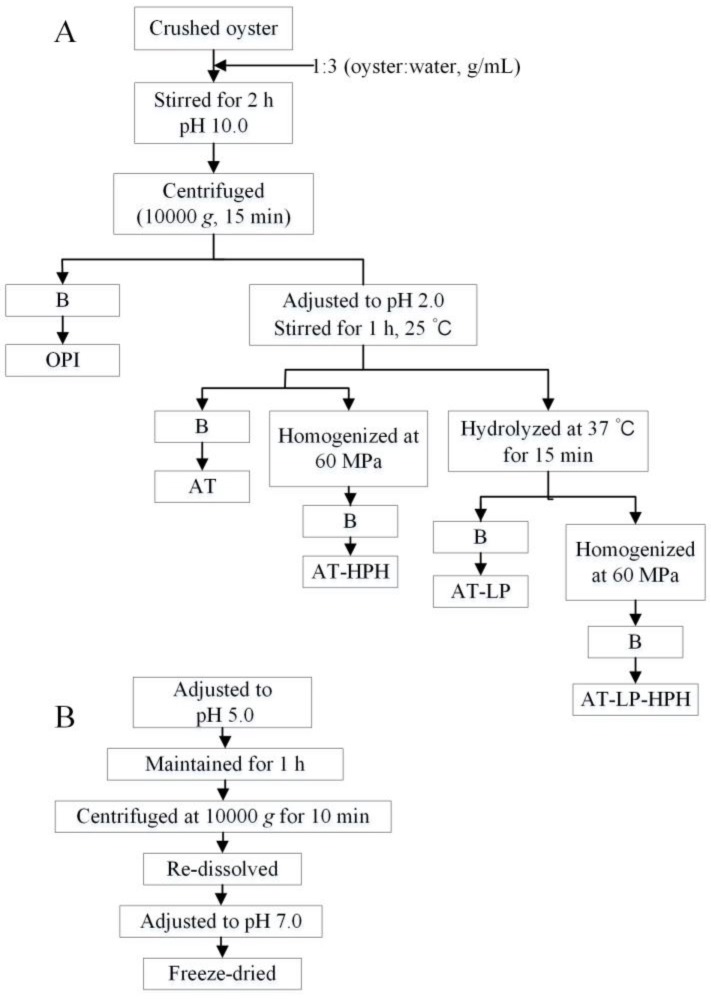
Scheme of sample preparation. Phase (**A**) and phase (**B**).

OPI was treated with acid, HPH, limited proteolysis with pepsin, and their combined treatments, as reported with some modifications [41,42]. Protein samples were prepared according to the process shown in Figure 6. Crushed oysters were dispersed in deionized water (1:3, *w*/*v*) and adjusted to pH 10.0 with 1 M NaOH. The mixture was stirred for 2 h at 25 °C and centrifuged at 10,000× *g* for 15 min. The supernatant was divided into two parts. One part was adjusted to pH 5.0 with 1 M HCl, maintained for 1 h, and centrifuged at 10,000 g for 10 min. The precipitate was re-dispersed in deionized water, neutralized to pH 7.0 using 1 M NaOH, and then freeze-dried (Figure 6B). The other part of the supernatant was adjusted to pH 2.0 with 1 M HCl, and stirred for 1 h at 25 °C, fractionated to four parts, and treated according to the following procedures (Figure 6A).

Acid treatment (AT): the pH 2.0 solution was treated following Figure 6B.

Acid treatment combined with high-pressure homogenization (AT-HPH): the pH 2.0 solution was homogenized at a pressure of 60 MPa for three cycles, and treated following Figure 6B.

Acid treatment combined with limited proteolysis treatment (AT-LP): the pH 2.0 solution was hydrolyzed for 15 min using pepsin (0.3:100, g/g protein) at 37 °C, and treated following Figure 6B.

Acid, limited proteolysis, and high-pressure homogenization treatment (AT-LP-HPH): after AT-LP treatment, the solution was treated by HPH at 60 MPa for three cycles, and treated following Figure 6B.

### 3.4. Determination of Degree of Hydrolysis (DH)

DH was quantified using the 2,4,6-trinitrobenzenesulfonic acid (TNBS) method as reported [43]. SDS (1%, *w*/*v*) was used to dilute samples and standard solution. Then 200 μL of dilutions was added into 2.0 mL of sodium phosphate buffer (pH 8.2). TNBS solution (0.1%, 2.0 mL) was added to the mixture, and incubated for 2 h at 50 °C in dark. Then 2.0 mL of 1 M HCl was added to stop the reaction. Samples were measured at 340 nm using an auto-microplate reader (Infinite M200, Tecan, Grödig, Austria). The standard curve was obtained with l-leucine. DH was calculated as:

(1) $$\mathrm{DH}\;\left(\%\right)=\frac{N}{N_{pb}}\times 100\%$$

where N is the amino nitrogen content of the sample after hydrolysis and *N_pb_* is the nitrogen content of the peptide bonds in the protein substrate, calculated according to the value of total nitrogen using the Kjeldhal method.

### 3.5. SDS-PAGE Analysis

SDS-PAGE was conducted using an electrophoresis system (Bio-Rad Laboratories, Hercules, CA, USA) as reported [44]. The acrylamide concentrations of separating gel and the stacking gel were 10% and 5%, respectively. Briefly, protein samples were dissolved in SDS-PAGE sample loading buffer, 5× (Beyotime Institute of Biotechnology, Haimen, China), to a final concentration of 2 mg/mL, and then heated at 100 °C for 10 min. Aliquots (10 μL) of samples were subjected to electrophoresis. After electrophoresis, Coomassie Brilliant Blue R-250 was used to stain gels. The molecular weights of bands were analyzed using QuantityOne-software (Bio-Rad, Hercules, CA, USA).

### 3.6. Determination of Protein Solubility

Protein solubility was determined according to a previous report with some modifications [45]. Protein samples was dispersed in deionized water (1%, *w*/*v*), adjusted to pH 3–10 with 1 M HCl or NaOH, and stirred for 1 h at 25 °C. The solution was centrifuged at 10,000× *g* for 15 min. The biuret method was used to determine the protein content. Bovine serum albumin (BSA) was used as the standard [46]. Protein solubility was calculated as:

(2) $$Solubility\;\left(\%\right)=\frac{supernatant\;protein\;concentration}{sample\;protein\;concentration}\times 100$$

### 3.7. Determination of Particle Size Distribution And Zeta Potential

Determination of particle size distribution and zeta potential was conducted using a Zetasizer 3000 (Malvern Instruments, Malvern, UK) as reported [47]. Briefly, protein samples were dissolved in deionized water (2 mg/mL), stirred for 1 h, and centrifuged at 3000× *g* for 3 min. After centrifugation, supernatant was diluted to 0.1 mg/mL and 1 mg/mL to measure particle size distribution and zeta potential, respectively.

### 3.8. Determination of Surface Hydrophobicity (H_0_) 

*H*_0_ was detected with 1-anilino-8-naphthalenesulfonate (ANS) as reported [48]. Protein samples were dissolved in 0.01 M sodium phosphate (pH 7.2), stirred for 1 h at 25 °C, and centrifuged at 10,000× *g* for 10 min. Supernatant was diluted to 0.1, 0.2, 0.4, 0.6, 0.8, and 1.0 mg/mL. Then 20 μL of ANS (8.0 mM) was added to 4 mL of protein solution. Fluorescence intensity (FI) was measured at an excitation wavelength of 390 nm and an emission wavelength of 470 nm using a fluorescence spectrophotometer (F-2700, Hitachi, Tokyo, Japan). The initial slope of FI versus protein concentration was calculated by linear regression analysis and used as an index for *H*_0_.

### 3.9. Determination of Foaming Properties

Foaming capacity (FC) and foaming stability (FS) were detected as reported with some modifications [49]. Briefly, protein samples were dissolved in 25 mL of deionized water (4%, *w*/*v*), placed into a 100-mL graduated cylinder, and then homogenized using a high-speed homogenizer (Ultra-Turrax T25, IKA Labor-technik, Staufen, Germany) at 10,000 rpm for 1 min. FC was calculated as:

(3) $$\mathrm{Foam}\;\mathrm{capacity}=\frac{V_{0}-25}{25}\times 100\%$$

(4) $$\mathrm{Foam}\;\mathrm{stability}=\frac{V_{30}-25}{V_{0}-25}\times 100\%$$

where *V*_0_ is the aqueous phase volume at 0 min, and *V*_30_ is the aqueous phase volume remaining after 30 min.

### 3.10. Determination of Emulsifying Properties

Emulsifying activity index (EAI) and emulsifying stability index (ESI) were detected as reported with modifications as described below [50]. Protein was dissolved in 30 mL of deionized water to 1% (*w*/*v*). Then 10 mL of corn oil was added and homogenized using a high-speed homogenizer (Ultra-Turrax T25, IKA Labor-technik, Staufen, Germany) at 10,000 rpm for 1 min. Aliquots (50 μL) of emulsion were taken from the bottom of the container, immediately (0 min) or 10 min after homogenization, then diluted in 5 mL of SDS (0.1%, *w*/*v*). The absorbance was read at 500 nm using a spectrophotometer (UV 2400, SOPTOP, Shanghai, China) after shaking for 5 s. EAI and ESI were calculated as:

(5) $$\mathrm{EAI}\;\left(m^{2}/g\right)=\frac{2\times 2.303\times A_{0}\times DF}{c\times \emptyset \times \left(1-\theta \right)\times 10000}$$

(6) $$\mathrm{ESI}\;\left(\min\right)=\frac{A_{0}}{A_{0}-A_{10}}\times 10$$

where *A*_0_ and *A*_10_ are the absorbance of the emulsions at 0 and 10 min, respectively. *DF* is the dilution factor, c is the initial concentration of protein (g/mL), ∅  is the optical path, and θ is the fraction of oil.

### 3.11. Statistical Analysis

All experiments were conducted in triplicate except the preparation of OPI and the modified samples. Data were then subjected to one-way ANOVA by means of the software SPSS 18.0. The mean comparison was made using the multiple ranges Duncan’s test (*p* < 0.05).

## 4. Conclusions

Acid treatment led to the dissociation and unfolding of OPI, while subsequent treatment such as limited proteolysis, HPH, and their combination remarkably improved the functional properties of OPI. Acid treatment produced flexible aggregates, as well as reduced particle size and solubility. On the contrary, limited hydrolysis increased the solubility of OPI. Furthermore, HPH enhanced the effectiveness of the above treatments. The emulsifying and foaming properties of acid- or hydrolysis-treated OPI significantly improved. In conclusion, a combination of acid treatment, limited proteolysis, and HPH improved the functional properties of OPI.

## Figures and Tables

**Table 1:** Protein solubility of OPI and the modified samples at different pH values.

Samples	pH Levels							
	3	4	5	6	7	8	9	10
OPI	54.4% ± 1.3% ^d^	3.9% ± 0.6% ^a^	2.6% ± 0.6% ^a^	7.6% ± 1.4% ^a^	27.1% ± 0.7% ^b^	27.4% ± 1.4% ^b^	41.5% ± 1.3% ^b^	59.8% ± 0.5% ^c^
AT	41.5% ± 0.8% ^b^	4.1% ± 0.7% ^a^	2.3% ± 0.5% ^a^	6.5% ± 1.3% ^a^	19.9% ± 0.8% ^a^	24.4% ± 1.5% ^b^	35.3% ± 1.1% ^a^	49.3% ± 0.6% ^a^
AT-HPH	31.6% ± 0.8% ^a^	4.1% ± 1.0% ^a^	2.9% ± 0.9% ^a^	7.4% ± 1.0% ^a^	20.6% ± 1.1% ^a^	27.3% ± 1.2% ^a^	40.1% ± 1.1% ^b^	53.8% ± 0.7% ^b^
AT-LP	32.6% ± 1.3% ^a^	11.6% ± 0.9% ^b^	7.7% ± 0.9% ^b^	7.5% ± 1.2% ^a^	27.4% ± 0.9% ^b^	30.6% ± 0.5% ^c^	53.6% ± 1.1% ^c^	73.7% ± 1.1% ^d^
AT-LP-HPH	52.3% ± 1.4% ^c^	15.7% ± 1.1% ^c^	11.8% ± 1.0% ^c^	15.8% ± 1.9% ^b^	31.7% ± 1.4% ^c^	42.6% ± 1.3% ^d^	61.4% ± 1.2% ^d^	91.7% ± 1.0% ^e^

Different letters represent significant differences at *p* < 0.05.

**Table 2:** *H*_0_ of OPI and the modified samples in 0.01 M phosphate buffer (pH 7.0).

Protein Samples	*H*_0_-ANS
OPI	402.08 ± 21.02 ^b^
AT	469.60 ± 18.59 ^c^
AT-HPH	563.04 ± 14.59 ^d^
AT-LP	314.92 ± 8.64 ^a^
AT-LP-HPH	335.40 ± 4.61 ^a^

Different letters represent significant differences at *p* < 0.05.
